# Thanking *Cancer Cell International’s* 2013 reviewers

**DOI:** 10.1186/1475-2867-14-11

**Published:** 2014-02-04

**Authors:** Denys Wheatley

**Affiliations:** 1 Director of BioMedES, Aberdeen, UK

## Abstract

**Contributing reviewers:**

The Editor-in-Chief would like to thank all the reviewers who contributed to the journal in 2013.

## 

Adrian Britschgi

Switzerland

Ai-ling Tian

China

Ajay Bommareddy

United States of America

Alberto Ferlin

Italy

Alessia Ongaro

Italy

Alexander Dobrovic

Australia

Alfredo De Paula

Brazil

Alok Mishra

United States of America

Amanda Ward

United States of America

Amanda Dixon-McIver

New Zealand

Ammad Farooqi

Pakistan

Anália Carmo

Portugal

Andrea Rasola

Italy

Andreas Villunger

Austria

Anna Litwiniec

Poland

Anna Friedl

Germany

Anthony Gavalas

Greece

Anthony Gavalas

Germany

Antoinette Hollestelle

Netherlands

Antonella Canini

Italy

Anwar Hossain

United States of America

Aras Emre Canda

Turkey

Ariana Bruzzone

Argentina

Ashraf Abdel-Naim

Saudi Arabia

Axel Hegele

Germany

Baoan Chen

China

Basavaraj Koti

India

Bassel El-Rayes

United States of America

Ben Loos

South Africa

Benedetta Bussolati

Italy

Bilge Sener

Turkey

Bin Shan

United States of America

Bo Cao

United States of America

Bo Guang Fan

Canada

Bonhong Min

Korea, South

Boris Zhivotovsky

Sweden

Boris Negrutskii

Ukraine

Boudewijn J Braakhuis

Netherlands

Bruno Robbs

Brazil

Bruno Vincenzi

Italy

Bulent Ozpolat

United States of America

Byong Chul Yoo

Korea, South

Cameron Bracken

Australia

Cecilia Hegardt

Sweden

Cedric Rebe

France

Chandramu Chetty

United States of America

Chin-Cheng Su

Taiwan

Christer Thomsen

Sweden

Christian Stock

Germany

Christiane Maier

Germany

Daigo Sumi

Japan

Daisuke Ennishi

Canada

Daniel Ramon Ciocca

Argentina

Daniela Gallo

Italy

Daniele Vergara

Italy

Darell Bigner

United States of America

David Buxton

United States of America

David Emlet

United States of America

Degang Shi

China

Delia Mezzanzanica

Italy

Delong Liu

United States of America

Deodutta Roy

United States of America

Dick Mosser

Canada

Dihua Shangguan

China

Dominique Modrowski

France

Dongjun Peng

United States of America

Douglas Demetrick

Canada

Douglas Hurst

United States of America

Dustin Deming

United States of America

Elena Takano

Australia

Elena Nadezhdina

Russian Federation

Elif Damla Arisan

Turkey

Elizabeth Guo

China

Ellen Heitzer

Austria

Elsa Bronze-da-Rocha

Portugal

Emilio Gonzalez

Spain

Erhong Meng

United States of America

Esra Erdal

Turkey

Eumorphia Konstantakou

Greece

Ewa Balcerczak

Poland

Fabio Luis Forti

Brazil

Fan Yi

China

Fang Yan

United States of America

Farah Khan

India

Federica Sotgia

United Kingdom

Fiona Yull

United States of America

Francois Martin

France

Frank Greco

United States of America

Fraz Awan

Pakistan

Gabriella D'Orazi

Kazakhstan

Gabriella Pagnan

Italy

Galatea Kallergi

Greece

Gangadhara Sareddy

United States of America

Gar-Yang Chau

Taiwan

Geert Bultynck

Belgium

George Calin

United States of America

Georgios Nteliopoulos

United Kingdom

Gian Luigi Russo

Italy

Giulio Metro

Italy

Gloria Calaf

United States of America

Grigori Rychkov

Australia

Guixing Qiu

China

Gulgun Oktay

Turkey

Guochang Hu

United States of America

Guoyou Zhang

Australia

Haiteng Deng

China

Hans-Joachim Mueller

Germany

Hark Kim

Korea, South

Hecheng Li

China

Helfrid Hochegger

United Kingdom

Hong Fan

China

Hong Chang

Canada

Hong He

United States of America

Hong Yang

China

Hong He

Australia

Hong Zheng

United States of America

Hongmei Nan

United States of America

Hong-Sheng Zhang

China

Hongwei Zhang

China

Hongwei Li

China

Huifang Hao

Japan

Ido Wolf

Israel

Indu Kohaar

United States of America

Irfan Asangani

United States of America

Isabel Andujar

Spain

Isabelle Dhennin-Duthille

France

Istvan Zupko

Hungary

Istvan Vermes

Netherlands

James A McCaul

United Kingdom

Jamie Jarboe

United States of America

Jean Koff

United States of America

Jekaterina Erenpreisa

Latvia

Jenifer Prosperi

United States of America

Jessy Deshane

United States of America

Jia Xu

United States of America

Jiahua Zhu

United States of America

Jiajun Shi

United States of America

Jing Lin

United States of America

Jiri Vrba

Czech Republic

Joana Carvalho

Portugal

John Pulikkan

Germany

Jong Bin Kim

Korea, South

Jong Kuk Park

Korea, South

Jun Yao

China

Jun Yasuda

Japan

Jun Yang

United States of America

Junaid Abdulghani

United States of America

June Zou

United States of America

Junming Yue

United States of America

Junming Guo

China

Justine Rudner

Germany

Kajsa Paulsson

Sweden

Kaleeckal Harikumar

United States of America

Kameswaran Ravichandran

United States of America

Karine Breckpot

Belgium

Karl Chai

United States of America

Katharine Irvine

Australia

Kazuhiro Kunimasa

Japan

Keizo Takenaga

Japan

Ken Young

United States of America

Ken-ichi Ogawara

Japan

Kevin Sun

New Zealand

Konradin Metze

Brazil

Kotb Abdelmohsen

United States of America

Kwadwo Asamoah Kusi

Ghana

Li Xiaorong

China

Li Cao

United States of America

Liguang Chen

United States of America

Ling Wang

China

Liping Liu

China

Lisa Murphy

Ireland

Lizong Shen

China

Ljubica Vucicevic

Serbia

Long-Cheng Li

United States of America

Lorenzo Sempere

United States of America

Luc Dirix

Belgium

Lucia Sfondrini

Italy

Luis Goya

Spain

Lyuba Varticovski

United States of America

Magda hagras Saudi

Arabia

Makoto Makishima

Japan

Manxiang Li

China

Marco Cerbón

Mexico

Maria Teresa Teresa

Italy

Marius Raica

Romania

Martin Liu

United States of America

Martino Introna

Italy

Maryna de Kock

South Africa

Massimo Derenzini

Italy

Massimo Aglietta

Italy

Massimo Zollo

Italy

Mayuko Furuta

Japan

Mehdi Shafa

Canada

Meixia Zhang

China

Meng Qingbing

China

Meng-Ru Shen

Taiwan

Michael Chan

Taiwan

Michael Graner

United States of America

Michele Caraglia

Italy

Mike Fainzilber

Iran

Ming Yuan

United Kingdom

Minghua Wu

China

Mitsuho Onimaru

Japan

Mohamed Saleh Mahmoud

Canada

Mohit Sachdeva

United States of America

Mu Zhang

United States of America

Mugdha Khaladkar

United States of America

Munirathinam Gnanasekar

United States of America

Muriel Priault

France

Muthusamy Kunnimalaiyaan

United States of America

Nahum Sonenberg

Canada

Natalia Ceaglio

Argentina

Natalija Eigeliene

Finland

Natarajan Aravindan

United States of America

Natasha Kyprianou

United States of America

Nathalie Johnson

Canada

Navasona Krishnan

United States of America

Nayoung Kim

Korea, South

Neelu Puri

United States of America

Nicholas Pavlidis

Greece

Nicola Aceto

United States of America

Nives Pecina-Slaus

Croatia

Nizar Mhaidat

Jordan

Ola Hermanson

Sweden

Oleh Stasyk

Ukraine

Oliver Treeck

Germany

Omar Mohafez

Japan

Osamu Miura

Japan

Pao-Ling Torng

Taiwan

Paolo Aurello

Italy

Paolo Martini

United States of America

Paolo Gandellini

Italy

Parvin Mehdipour

Iran

Patricio Aller

Spain

Paul Agutter

United Kingdom

Paul Bollyky

United States of America

Pavel Soucek

Czech Republic

Pedro P. Lopez-Casas

Spain

Peiman Liu

United States of America

Peiyu Pu

China

Penka Nikolova

United Kingdom

Periasamy Selvaraj

United States of America

Peter Schraml

Switzerland

Peter Olbert

Germany

Phyllis Wachsberger

United States of America

Pier Giorgio Petronini

Italy

Ping Zhu

China

Ping-an He

China

Po-Wu Gean

Taiwan

Qian Xu

China

Qing-Hua Meng

China

Qingsong Zhu

United States of America

Rajakishore Mishra

United States of America

Rajendra Sharma

Canada

Rakesh Naidu

Malaysia

Ramon Alemany

Spain

Ravindresh Chhabra

India

Rik Derynck

United States of America

Rishi Chhipa

India

Robert Milewski

Poland

Rohit Malik

United States of America

Roland Lauster

Germany

Romain Debret

France

Roman Eliseev

United States of America

Ruben Plentz

Germany

Sabine Hombach-Klonisch

Canada

Salvatore Pizzo

United States of America

Samer Hakim

Germany

Sander van den Driesche

United Kingdom

Sandra Karlsson

Sweden

Sarah Prior

United Kingdom

Saskia Cillessen

Netherlands

Sen Zhang

China

Shaofang Wu

United States of America

Sheng-mei Zhu

China

Shigeki Ito

Japan

Shounanyi Yi

Australia

Shuyu Ren

United States of America

Sidney Yu

Hong Kong

Smadar Avigad

Israel

Song YQ

China

Sreenivasulu Chintala

United States of America

Stefan Pusch

Germany

Stephan Reshkin

Italy

Stephanie Vispe

France

Steve Chambers

New Zealand

Steven Van Laere

Belgium

Sudhakar Veeranki

United States of America

Suoqin Tang

China

Suresh Tikoo

Canada

Sydney Mukaratirwa

United Kingdom

Tadashi Kondo

Japan

Tae il Kim

Korea, South

Takatsune Shimizu

Japan

Takeshi Miyamoto

Japan

Takuya Moriya

Japan

Tao Ren

China

Tao Lin

United States of America

Taodi Liu

China

Tatiana Zybina

Russian Federation

Terry Moody

United States of America

Thomas Scheper

Germany

Tiago Rodrigues

Brazil

Timothy Gershon

United States of America

Toni Ibrahim

Italy

Tonya Walser

United States of America

Tsui-Ling Chang

Taiwan

Tsutomu Shimura

Japan

Ugo Testa

Italy

Ulrich Renner

Germany

Ulrike Stein

Germany

Umberto Galderisi

Italy

Urmila Santanam

United States of America

Urs Lichtenauer

Germany

Valentina Todorova

United States of America

Valery Grdzelishvili

United States of America

Vishal Gupta

India

Vladimir Pushkarev

Ukraine

Vladimir Gogvadze

Sweden

W. Keith Miskimins

United States of America

Wanming Zhao

United States of America

Wei Du

United States of America

Wei Ding

China

Weili Cai

United States of America

Wen Zhuang

China

Wen Ying Wu

China

Wendy Hwang-Verslues

Taiwan

Wenjun Wang

China

Wenling Wang

China

Wenxi Wu

China

Wenyue Sun

United States of America

William Cho

Hong Kong

Wolfram Miekisch

Germany

Wu Dong-Cheng

China

Xiaobin Jia

China

Xiaobing He

United States of America

Xiaobo Li

United States of America

Xiaochun Xu

United States of America

Xiaohua Jiang

Hong Kong

Xiaoping Miao

China

Xiaoqing Niu

China

Xianoyong Zheng

United States of America

Xin-xia Tian

China

Xiuwu Zhang

United States of America

Yajuan Su

China

Yan Huang

United States of America

Yanfeng Qi

United States of America

Yang Yao

China

Yang-Chang Wu

Taiwan

Yangqiu Li

China

Yei-Tsung Chen

Singapore

Yen-Ni Teng

Taiwan

Yi Zhang

China

Yiannis Drosos

United States of America

Ying Jin

China

Yingpeng Liu

United States of America

Ymera Pignochino

Italy

Yoji Nagashima

Japan

Yong Yang

China

Yongwen Chen

China

Yoshiki Murakami

Japan

Yoshio Honma

Japan

Yu Liu

United States of America

Yu Jin

United States of America

Yunge Zhao

United States of America

Zahid Siddik

United States of America

Zeyi Zheng

United States of America

Zhaoxiang Bian

Hong Kong

Zhi Wang

China

Zhi-guang Tu

China

Zhi-Hong Wen

Taiwan

Zhijun Dai

China

Zihua Zeng

United States of America

Zongbing You

United States of America

